# Low molecular weight 35 kDa hyaluronan fragment HA35 in the treatment of bone metastasis pain: A case report

**DOI:** 10.1097/MD.0000000000039145

**Published:** 2024-08-02

**Authors:** Zongchun Zhang, Xiaoxiao Jia, Dylan Treger, Mizhou Hui

**Affiliations:** a Department of Radiotherapy, Qingdao Cancer Hospital, Qingdao, China; b Department of anesthesiology, Hynaut Laboratories, Hynaut Group, Qingdao, China; c DeWitt Daughtry Family Department of Surgery, Miller School of Medicine, University of Miami, Miami, FL; d Pharmaceutical Manufacture Department, Nakhia Impex Pharmaceutical Co., Ltd., Ulaanbaatar, Mongolia.

**Keywords:** 35 kDa hyaluronan fragments, bone metastasis pain, cancer pain, HA35, pain relief

## Abstract

**Rationale:**

Late-stage cancer patients often experience severe pain due to bone metastasis, caused by structural damage and cancer-induced inflammation. Hyaluronan, known to alleviate pain by blocking the TRVP1 calcium channel, faces limitations due to its high molecular weight. However, 35 kDa low molecular weight hyaluronan fragment (HA35) have shown promise in relieving various pains, including cancer-related pain. Nonetheless, evidence regarding their efficacy in bone metastasis pain remains scarce.

**Patients concerns:**

A 52-year-old female with a rectal malignant tumor and multiple secondary tumors in the sacrum and lungs, accompanied by bone metastasis pain. Despite undergoing radiotherapy, her pain relief was unsatisfactory. Before treatment with HA35, her numerical rating scale score was 10, severely affecting her sleep, appetite, and daily activities.

**Diagnoses:**

The patient was diagnosed with rectal malignant tumor with multiple metastases, presenting symptoms such as sacral metastasis pain, anal pain, lower limb pain, and anterior abdominal pain. Sacral metastasis pain and lower limb pain indicated a clear diagnosis of bone metastasis pain.

**Interventions:**

Treatment involved subcutaneous injection into the deep fat tissue layer of the abdomen. A subcutaneous injection of 100 mg/5 mL of HA35 was administered once into the deep fat tissue of the abdomen, with subsequent injections repeated every 3 days.

**Outcomes:**

Following 1 injection, the patient’s pain score decreased to 6 points within 20 minutes, providing 40% pain relief. After 40 minutes, the score further dropped to 4 points, with 60% pain relief. After 50 injections, pain was consistently controlled at around 3 points.

**Lessons subsections:**

Subcutaneous injection of HA35 into the abdominal fat tissue effectively alleviates pain in cancer and bone metastasis patients resistant to conventional treatments. Additionally, it helps alleviate anxiety and fatigue, and improves diet and sleep, thereby offering crucial palliative care for advanced cancer patients.

## 1. Introduction

Bone metastasis pain represents a significant challenge for cancer patients in advanced stages of the disease. Originating from both structural damage to bones and inflammation induced by cancer cells,^[1]^ this pain often imposes substantial suffering. Occasionally, bone fractures may exacerbate the pain. While complete cure of bone metastases remains elusive, treatment strategies aim to impede further cancer progression and alleviate associated symptoms, particularly bone pain.^[2]^ In this context, we outline the advantages and drawbacks of commonly employed treatments.^[3–6]^

Corticosteroids frequently serve as adjunct therapy for cancer-related pain, including that from bone metastases.^[3]^ However, prolonged corticosteroid use entails considerable adverse effects, notwithstanding the perceived benefits outweighing the risks upon risk–benefit analysis.^[4]^ Opioids constitute another cornerstone in managing bone metastasis pain,^[5]^ offering enduring analgesic effects, with a majority of patients experiencing relief or pain management. Yet, noteworthy side effects such as physiological dependence, tolerance, addiction, sedation, constipation, nausea, vomiting, and respiratory depression can curtail their sustained utility. Prolonged opioid therapy may even lead to a noticeable reduction in analgesic efficacy, irrespective of pain progression. Bisphosphonates also find application in alleviating bone metastasis pain.^[6]^ However, these agents have not emerged as the most efficacious treatment method for mitigating pain stemming from cancer metastases. Thus, given the enigmatic molecular underpinnings of bone metastasis pain, the side effect profiles and tolerability of conventional pharmacotherapies often inadequately address this form of pain.

In addition to the aforementioned treatment modalities, radiotherapy stands out as the most effective intervention for assuaging bone metastasis pain.^[7]^ Furthermore, denosumab, a monoclonal antibody therapy drug originally intended for giant cell tumors, has garnered approval for bone metastasis pain management.^[8]^ Notably, our study presents a case involving the utilization of hyaluronan fragments for bone metastasis pain management, proposing an innovative therapeutic avenue for ameliorating cancer-related bone metastasis pain via hyaluronan fragments. Research suggests that 35 kDa low molecular weight hyaluronan fragments (HA35) efficaciously address various pain types, encompassing inflammation,^[9]^ cancer,^[10]^ wounds,^[11]^ and radiation-induced pain. This report is a special case in the clinical study of radiotherapy for colorectal cancer (NCT06209970).^[10]^

## 2. Case presentation

This study was approved by a constituted review board (Ethics Committee of the Qingdao Cancer Hospital). This study adhered to the principles outlined in the Helsinki Declaration and obtained written informed consent from patients and guardians.

### 2.1. Clinical information

#### 2.1.1. Patient

A 52-year-old patient was diagnosed with rectal malignant tumor, sacral secondary malignant tumor, and lung secondary malignant tumor in 2019, undergoing surgical intervention as part of the treatment. In 2022, tumor recurrence presented with coccygeal pain, leading to radiotherapy. Subsequent diagnoses in 2023 included a female pelvic abscess, rectovaginal fistula, anorectal fistula, and hypothyroidism, necessitating surgery followed by radiotherapy and traditional Chinese medicine. Laboratory tests in Table 1 for inflammatory markers (white blood cells, lymphocytes, C-reactive protein, etc) and related biochemical indicators mostly show abnormalities. Upon admission for this study, the patient received routine care and resumed normal activities post-radiotherapy, exhibiting no adverse symptoms.

**Table 1:** Laboratory tests reports.

Test item (abbreviation)	Result	Reference interval
White cell count (WBC)	18.21	3.50 to 9.50 * 10^−9^/L
lymphocytes percentage (LYM%)	3.60	20.00 to 40.00%
Red blood count (RBC)	3.19	3.80 to 5.10 * 10^−12^/L
Hemoglobin (HGB)	79.00	115.00 to 150.00 g/L
Hematocrit (HCT)	25.80	35.00 to 45.00%
C-reactive protein (CRP)	109.12	≤10.00 mg/L
Na (Na^+^)	125.60	137.00 to 145.00 mmol/L
CL (Cl^−^)	85.80	98.00 to 107.00 mmol/L
Total protein (TP)	58.90	60.00 to 85.00 g/L
Albumin (ALB)	26.30	35.00 to 55.00 mmol/L
Alanine aminotransferase (ALT)	9.00	7.00 to 40.00 U/L
Aspartate aminotransferase (AST)	12.00	13.00 to 35.00 U/L
Alkaline phosphatase (ALP)	51.00	35.00 to 135.00 U/L
Urea (Urea)	6.32	3.89 to 6.11 mmol/L
Uric acid (UA)	124.00	155.00 to 357.00 µmol/L

The patient reported after discharge that, in addition to the pain in the sacrococcygeal region caused by cancer metastasis, the pain from prolonged bed rest due to external pressure was also very severe. The severe pain has resulted in poor appetite and sleep for the patient, and her complexion appears unhealthy. Long-term oral administration of Luguike (acetaminophen dihydrocodeine) and tramadol was ineffective for pain relief, so it was switched to oral OxyContin, which also did not help.

### 2.2. Treatment

The patient was administered a subcutaneous injection into the abdomen, referred to as HA35, which contained a mixture of hyaluronidase (1500 units per vial, China Food and Drug Administration registration number H31022111, SPH No. 1 Biochemical & Pharmaceutical Co., Ltd.) and hyaluronan (10 mg/mL, China Food and Drug Administration Registration No. H20174089, Shanghai Haohai Biotechnology Co., Ltd.) A 100 mg dose was initially administered, with the patient’s numerical rating scale (NPRS) scores documented post. Subsequent injections were administered every 2 to 3 days based on pain recurrence, with adjustments made every 5 to 7 days thereafter. Oral pain medication and anti-inflammatory drugs were continued throughout treatment.

### 2.3. Pain assessment

Table 2 presents pain scores and effectiveness of pain relief, indicating reduction within 20 minutes postinjection and optimal efficacy at 40 minutes, lasting 2 to 3 days. Patient received injections every 3 days initially, with pain reduction observed after 16 injections, prompting adjustment to once every 4 days. After 30 injections, notable improvements in mental state, mobility, and tumor regression led to further reduction to once every 5 days. Patient received a total of 50 injections.

**Table 2:** Pain scores and assessments at different time points in patients.

	Treatment time	Pain numeric rating scale (NPRS)	Pain relief percentage	Pain assessment
After 1 injection	Before treatment	10 score	0%	The patient experienced severe pain in the lower abdomen and legs, bone pain resulting from lung cancer metastasis, and muscle compression pain in the legs due to prolonged bed rest. These symptoms were accompanied by sleepless nights, long-term loss of appetite, and inability to eat
	Treatment for 20 min	6 score	40%	Pain reduced by about 40%, leg pain began to ease
	Treatment for 40 min	4 score	60%	The overall pain decreased by approximately 60%, with significant relief in leg pain, enabling semiindependent movement and the ability to turn over. Additionally, thigh root pain caused by long-term compression was completely alleviated
	Treatment for 60 min	4 score	60%	The pain persists at its current level, but the patient is noticeably more alert and willing to change positions
	Treatment for 24 h	4 score	60%	The patient’s pain has not improved further, but overall comfort is satisfactory, and the patient is able to sleep at night
	Treatment for 3 d	9 score	10%	The pain is beginning to return
After 16 injections		4 score	60%	The patient feels relaxed overall, with pain not affecting their ability to lie down comfortably, and has a good appetite
After 30 injections		3 score	70%	The patient is in good condition, with a fair and improved complexion. Pain does not affect their ability to lie down comfortably, and they have a good appetite
After 50 injections		3 score	70%	The patient has transitioned from being bedridden for a long time to wanting to get up and walk

After administering 50 vials of the injection, we conducted a quality of life questionnaire for the patient. Table 3 shows the scores of the European Organization for Research and Treatment of Cancer Quality of Life Questionnaire-Core 30 (EORTC QLQ-C30). The results indicate that after completing all injections, there was an increase in the scores for the functional domains and overall health status over the past week. Scores for symptoms such as fatigue, nausea and vomiting, and pain, as well as other negative symptom indicators, decreased. Throughout the entire treatment process, the patient did not experience any of the side effects commonly associated with other oral pain medications.

**Table 3:** Quality of life scores before and after patient treatment.

Index	Scores before treatment	Scores after 50 injections
Physical function	13.3	46.7
Role function	33.3	66.7
Emotional function	16.7	75.0
Cognitive function	58.3	100
Social function	16.7	33.3
Overall health condition	16.7	75.0
Fatigue	77.8	33.3
Nausea and vomiting	33.3	16.7
Pain	100	33.3
Difficulty breathing	66.7	33.3
Insomia	100	33.3
Loss of appetite	100	33.3
Constipation	33.3	0
Diarrhea	66.7	0
Economic hardship	33.3	33.3

## 3. Discussion

Tumor bone metastasis entails the spread of malignant tumors from nonbone tissues to bone tissue through the bloodstream or, occasionally, via lymphatic channels.^[12]^ This process leads to the gradual formation of new tumor lesions, causing disruption to normal bone tissue and often demonstrating as severe pain. Clinically, bone metastatic pain is recognized as a significant complication of cancer, with statistics indicating that a substantial proportion of patients succumb to cancer-related complications rather than the cancer itself.^[1,13]^ In the case presented herein, the patient suffered from malignant rectal tumor, sacral metastatic malignant tumor, and lung metastatic malignant tumor. While radiotherapy initially provided some relief without impeding normal activities, disease recurrence exacerbated by the COVID-19 pandemic resulted in deteriorating condition. Neither analgesic medications nor continued radiotherapy adequately controlled the pain. In response to the patient’s unmet needs, we pursued treatment with injectable HA35.

HA35 is a low molecular weight fragment of hyaluronan generated by the enzymatic digestion of high molecular weight hyaluronan using recombinant human hyaluronidase PH20 or hyaluronidase PH20 extracted from bovine testes.^[14]^ It exhibits outstanding tissue penetrability and rapidly binds to cellular or tissue receptors.^[9,15]^ Clinical investigations have explained its therapeutic benefits in alleviating various forms of pain, including inflammatory and neuropathic pain in differ regions.^[10,11]^ Recent research, notably Caires’s study in Nature Communications, suggests that HA35’s pain-relieving effect may be intervened by the calcium channel TRPV1.^[15]^

The outcomes (Tables 2 and 3) presented in our study represent a significant advancement, showcasing the remarkable efficacy of HA35 when administered into the deep fat tissue layer of the abdomen for alleviating bone metastasis pain. Importantly, these findings emphasize HA35’s potential as a promising therapeutic intervention for pain management, as evidenced by the gradual reduction in medication dosage posttreatment and concurrent amelioration of associated symptoms, thereby enhancing overall quality of life.^[16]^ Comparative analysis with conventional medications commonly employed for bone metastasis pain relief highlights HA35’s favorable safety profile, positioning it as a compelling option for the treatment of bone metastasis-induced pain.^[17]^

## 4. Conclusion

Bone metastasis pain constitutes a crucial facet of palliative care for advanced cancer patients. 35 kDa low molecular weight hyaluronan fragment (HA35) emerge as a promising therapy for alleviating bone metastasis pain.

## Acknowledgments

We wish to express our gratitude to all the staff for their hard work and dedication in implementing the intervention and evaluation components of the study.

## Author contributions

**Conceptualization:** Zongchun Zhang, Mizhou Hui.

**Data curation:** Zongchun Zhang, Xiaoxiao Jia.

**Writing – original draft:** Xiaoxiao Jia, Mizhou Hui.

**Formal analysis:** Dylan Treger, Mizhou Hui.

**Writing – review & editing:** Mizhou Hui.
